# Elucidation of tRNA‐Derived Fragments in Alzheimer’s Disease: Cell‐Type Implication for New Biomarkers and Regulators

**DOI:** 10.1002/alz.091675

**Published:** 2025-01-09

**Authors:** Meagan D Rippee‐Brooks, Hannah Vedder, Wenzhe Wu, Jianli Dong, Miguel A Pappolla, Xiang Fang, Xiaoyong Bao

**Affiliations:** ^1^ University of Texas Medical Branch, Galveston, TX USA

## Abstract

**Background:**

Alzheimer’s disease (AD), the most common type of dementia, affects at least twenty‐four million people globally, yet, the causation, mechanisms of progression, and therapeutic strategies remain elusive. Currently, tRNA‐derived RNA fragments (tRFs), a family of recently discovered small non‐coding RNAs (sncRNAs), have surfaced as promising biomarkers for many diseases, including AD. Our work revealed that several AD‐impacted tRFs in human hippocampus, CSF, and serum.

**Method:**

Herein, we further characterized the expression of tRFs in AD through elucidating exactly which cell types of the brain contribute to the higher expression of tRFs, using human induced pluripotent stem cells (iPSCs)‐derived cells. We further delve into the identification of tRFs, commonly affected by emerging SARS‐CoV‐2 infection and AD, for a future study on the influence of SARS‐CoV‐2 infection on AD.

**Result:**

Our results indicate that neurons derived from AD iPSC have significantly enhanced tRF expression than neurons derived from healthy adult donor. The impacted tRFs include tRFs derived from the 5’‐end of mature tRNA encoding ProAGG (tRF5‐ProAGG), GluCTC (tRF5‐GluCTC), and GlyGCC (tRF5‐GlyGCC). Such a difference did not show in iPSC‐derived astrocytes. Interestingly, among AD‐impacted tRFs in neurons, two of them are also inducible by SARS‐CoV‐2 infection.

**Conclusion:**

tRF5‐ProAGG, ‐GluCTC, and ‐GlyGCC continue to show the potential as AD biomarkers originating from neurons from within the brain. Commonly impacted tRFs by SARS‐CoV‐2 and AD, combined with our recently published data showing the impact of tRF5 on the regulation of genes important for neuronal functions, implicates SARS‐CoV‐2’s influence on AD.

